# Gender difference of geographic distribution of the stroke incidence affected by socioeconomic, clinical and urban-rural factors: an ecological study based on data from the Brest stroke registry in France

**DOI:** 10.1186/s12889-020-10026-7

**Published:** 2021-01-06

**Authors:** Cindy M. Padilla, Anais Foucault, Olivier Grimaud, Emmanuel Nowak, Serge Timsit

**Affiliations:** 1 Univ Rennes, EHESP, REPERES (Recherche en pharmaco-épidémiologie et recours aux soins) – EA 7449, 15, Avenue du Professeur Léon Bernard, 35043 Rennes, France; 2 Centre d’Investigation Clinique-INSERM CIC 1412, CHRU, Brest, France; 3 Univ Brest, Inserm, EFS, UMR 1078, GGB, Neurology and Stroke unit Department, CHRU de Brest, Université de Bretagne Occidentale, Inserm 1078, Brest, F-29200 France

**Keywords:** Stroke, Socioeconomic factors, Urban rural, Incidence, geographically weighted regression (GWR), spatial variations

## Abstract

**Background:**

Mapping the spatial distribution of disease occurrence is a strategy to identify contextual factors that could be useful for public health policies. The purpose of this ecological study was to examine to which extent the socioeconomic deprivation and the urbanization level can explain gender difference of geographic distribution in stroke incidence in Pays de Brest, France between 2008 and 2013.

**Methods:**

Stroke cases aged 60 years or more were extracted from the Brest stroke registry and combined at the census block level. Contextual socioeconomic, demographic, and geographic variables at the census block level come from the 2013 national census. We used spatial and non-spatial regression models to study the geographic correlation between socioeconomic deprivation, degree or urbanization and stroke incidence. We generated maps using spatial geographically weighted models, after longitude and latitude smoothing and adjustment for covariates.

**Results:**

Stroke incidence was comparable in women and men (6.26 ± 3.5 vs 6.91 ± 3.3 per 1000 inhabitants-year, respectively). Results showed different patterns of the distribution of stroke risk and its association with deprivation or urbanisation across gender. For women, stroke incidence was spatially homogeneous over the entire study area, but was associated with deprivation level in urban census blocks: age adjusted risk ratio of high versus low deprivation = 1.24, [95%CI 1.04–1.46]. For men, three geographic clusters were identified. One located in the northern rural and deprived census blocks with a 9–14% increase in the risk of stroke. Two others clusters located in the south-eastern (mostly urban part) and south-western (suburban and rural part) with low deprivation level and associated with higher risk of stroke incidence between (3 and 8%) and (8.5 and 19%) respectively. There were no differences in profile of cardiovascular risk factors, stroke type and stroke severity between clusters, or when comparing clusters cases to the rest of the study population.

**Conclusions:**

Understanding whether and how neighborhood and patient’s characteristics influence stroke risk may be useful for both epidemiological research and healthcare service planning.

## Background

Stroke remains a devastating disease in Europe despite major therapeutic improvements in the recent decades. At the beginning of the twenty-first century, the age-standardized stroke incidence in Europe ranged from 95 to 290 individuals per 100,000 inhabitants-year. However, the expected number of patients who will have a stroke each year will inevitably continue to rise due to population ageing. In France, in 2014, the age-standardized rate of hospital stroke admission was 167.9 per 100,000 and was 1.5 times higher in men than in women [1]. Stroke constitutes the first cause of mortality among women and the third among men, the age-standardized in hospital mortality rate was 14.3% in 2014. Moreover, it is a leading cause of serious disability. A study by the French national health insurance database suggests that in 2013 the cost of stroke care amounted to 3.5 billion € [2].

Stroke incidence is influenced by demographic, socioeconomic status (SES), and geographic factors [3–7]. Previous studies have investigated the association between stroke incidence and contextual SES level alone or after controlling for individual SES characteristics [8]. They found higher risk of stroke in groups with lower income [9–11] and education levels [12, 13], and in groups with higher deprivation levels [6, 14–17]. Epidemiologic studies reveal a clear age-by-sex interaction in stroke prevalence, incidence, and mortality [18].

In France, previous studies showed that the Brittany region (western part of France) had the highest ischemic stroke incidence in 2014 (137.9/100,000) [19], and demonstrated geographic variations with one cluster of high incidence in Brittany [20]. Level of access to primary care could determine these geographic variations. Indeed, stroke clustering in specific areas might be partly explained by the influence of material infrastructures and healthcare services, behavior and healthcare consumption [21, 22]. In 2016, a meta-analysis showed that poor social support was associated with a 32% increase in the risk of stroke [23], and Otto et al. suggested that social support is related to the urbanization level of the residential neighborhood [15].

Most stroke registries are based in urban settings. To our knowledge the French Brest Stroke Registry provides a unique opportunity to identify variations in stroke incidence along the rural-urban gradient. Indeed, it is important to understand whether and how neighborhood characteristics influence the stroke risk in order to develop policies for its prevention and management in urban and also in remote rural areas [13, 24]. The objectives of this study were. i) To analyse the spatial distribution of stroke incidence at the census block level in Pays de Brest. ii) To examine whether stroke incidence is associated with contextual determinants as socioeconomic deprivation and urbanization degree. iii) To assess whether patient characteristics varied inside and outside high-incidence stroke clusters when detected. Because of evidence of differences in the physiopathology and epidemiology of stroke across gender, we explore these questions separately in men and women [18].

## Methods

### Study setting

**Fig. 1:**
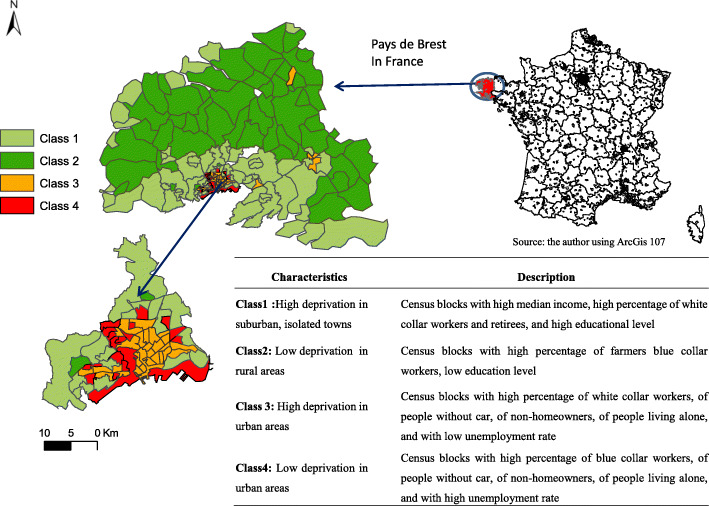
Description of the four deprivation & urban-rural index classes and their geographic distribution in the Pays de Brest administrative region, France. Maps were created by the authors using ArcGIS software

The study is an ecological study based on the area covered by the Brest Stroke Registry (BSR) which includes 79 municipalities. The BSR is set in the French Pays de Brest administrative region, (Fig. 1) located in the western part of Brittany. This area is of particular interest because it contains both rural and urban areas and their urban landscapes are contrasted in terms of certain significant demographic and socioeconomic characteristics.

The municipalities within the study area are subdivided in 174 census blocks which represents the spatial unit of analysis in this study. Census blocks are small geographic areas that contain around 2000 inhabitants. These statistical entities are designed to be relatively homogeneous in terms of population characteristics, socioeconomic status and living conditions [25].

### Data collection

#### Stroke cases

For the present ecological study, we included all incident cases of ischemic and hemorrhagic stroke that occurred in ≥60-year-old registered in the BSR between 1 January 2008 and 31 December 2013. Cases were aggregated at the census block level using their home address. We decided to focus on cases aged 60 years or more because very few stroke occurs in the younger active population and their inclusion was likely to increase statistical noise in an analysis at census block level. The Brest Stroke Registry is an ongoing prospective community-based stroke registry covering a population of 366,000 inhabitants. Since 2008, it operates using multiple information sources for the identification of stroke cases: public and private hospitals, radiology clinics that perform brain imaging, neurologists, and general practitioners. A validation committee confirms the identified cases. An evaluation of BSR performance based on capture-recapture analysis suggested a level of completeness in excess of 90% [26]. Two definitions of stroke cases were considered. The used ICD-10 codes were I63 (cerebral infarction) and I64 (stroke, not specified): 1) new focal neurological deficit with symptoms and signs coherent with a diagnosis of stroke, according to the World Health Organizations criteria, lasting for more than 24 h or death in the first 24 h; 2) all neurological focal deficits lasting at least 1 h or resolving within 1 h, but with abnormal brain imaging associated with a clinically relevant picture.

Patients or their legal representatives gave their written informed consent for participation. This study was approved by the local ethics committee. Access is subjected to current data protection guidelines compliance and prior approval from the national agency for data protection: “Comité consultatif sur le traitement de l’information en matière de recherche” and “Commission nationale informatique et liberté”.

Data extracted from the Brest Stroke Registry to compare the characteristics of patient residing inside and outside high-incidence stroke clusters were: sociodemographic data (age and sex); clinical data, including stroke type (ischemic or hemorrhagic), stroke severity (National Institute of Health Stroke Score (NIHSS) score < 6, 6–13, > 13); presence of cardiovascular risk factors before stroke (high blood pressure, cardiac arrhythmia, diabetes, and dyslipidemia). Missing values, which represent 11% for severity and less than 4% for all patients regarding risk factors; were not taking into account in the analysis.

#### Socioeconomic and urban-rural contextual effects

First, the level of deprivation for each census block was estimated using the French deprivation (FDep) index [27]. This index was defined and evaluated in previous studies that investigated environmental and health inequalities [19]. It was built by performing a Principal Components Analysis (PCA) using four variables: average household income, percentage of high school graduates in the ≤15-year-old population, percentage of workers in the active population, and unemployment rate, from the French national census of 2013 collected by the French National Institute for Statistics and Economic Studies (INSEE).

Then, a deprivation & urban-rural index was generated. The deprivation & urban-rural index was built using the same method as for the FDep index: PCA and the same four variables with the addition of a new urban-rural variable with four classes (urban, suburban, isolated town, and rural) depending on the number of towns and inhabitants, using INSEE data (Fig. 1).

### Statistical analysis

#### Stroke incidence risk and standardization

To assess stroke frequency and its spatial distribution in the different census blocks, the age-standardized stroke incidence risk with 95% confidence intervals was computed using the indirect standardization method [28]. This procedure ensured that differences in the geographic distribution of the stroke incidence risk were not influenced by geographic variations in the age distribution of the population. The stroke incidence risk was computed as the observed number of stroke cases in all > 60-year-old individuals during each age-period divided by the expected cases, reported per census block. The data on population (by sex and age period) came from INSEE. The age-standardized stroke incidence risks were presented per 1000 inhabitants-year.

#### Ordinary Poisson regression and geographically weighted Poisson regression models

First, potential associations between stroke incidence risk and contextual socioeconomic deprivation and urban-rural level were investigated separately for men and women using regression models. Data are fitted using the generalized linear model function with a Poisson distribution which is suitable for dealing with numerical data from a small area. As significant overdispersion was detected in the models for women, Poisson regression was inappropriate, and negative binomial models were used for all the other analysis in this group. Second, Koenker (BP) statistic was used to determine if spatial variability exist in these associations. When the *p* value was high, the assumption of stationarity in the relation throughout the study area was verified, so the ordinary Poisson or negative binomial model fitted better the dataset. Alternatively, when the *p* value was low (*p* < 0.10) suggesting evidence of spatial variability of the associations, the more suitable Geographic Weighted Regression method was used.

When there is not a stationarity in the relation throughout the study area, a local GWR analysis was carried out. The aim is to determine the existence of census block clusters with higher or lower risk of stroke incidence. Local GWR models allowed estimating as many local regression coefficients as the number of locations in the study area. For the local Poisson GWR, this model was built as follows:

$$ {\displaystyle \begin{array}{l}\mathrm{Local}\kern0.17em \mathrm{Poisson}\;\mathrm{GWR}:\mathrm{y}=\mathrm{Poisson}\;\left[\exp \left(\upeta \mathrm{i}\right)\right]\\ {}\upeta \mathrm{i}={\sum}_{\mathrm{k}}\upbeta \mathrm{k}\;\left(\mathrm{Ui},\mathrm{Vi}\right)\;{\mathrm{X}}_{\left(\mathrm{k},\mathrm{i}\right)}={X}_i^t\;\upbeta \left(\mathrm{Ui}\right)\end{array}} $$

where Χ(k,i) and βk are the kth explanatory variable and its local regression coefficient that is unique to location U, respectively. Thus, the regression coefficients vary based on the spatial location Ui = (ui, vi). The expected response value of the ith observation, E[yi], is related to the linear predictor via a Poisson link function.

To calibrate this equation, a bi-square adaptive weighting kernel function was used to account for the spatial structure (density, shape, and size of the census blocks), and the appropriate bandwidth was selected using the golden section method [29]. Locations near i have a stronger influence in the estimation of βj(ui,vi) than locations away from i. In the GWR model, localized parameter estimates can be obtained for any location i, which in turn allows the creation of a map showing a continuous surface of parameter values and the investigation of the spatial variability (non-stationarity) of these parameters. Additionally, the corrected Akaike Information Criterion (AICc) and the pseudo-adjusted coefficient of determination (adjusted R^2^) were used as goodness of fit estimation for model comparison. The GWR models were produced with R using GWmodel [30] (R Development Core Team, 2011). All map layouts were created using ArcMap v. 10.5 from ESRI.

## Results

### Spatial distribution of the stroke risk

We were provided with a dataset for 3870 stroke cases extracted. Of these, those who have less than 60 years old were excluded (782 cases). The number of incident stroke cases aggregated at the census block level was 3088 (1743 women and 1345 men).This corresponds to an incidence of 6.67 strokes per 1000 inhabitants-year in Pays de Brest over the period 2008–2013. Although stroke incidence was comparable in women and men (6.26 ± 3.5 vs 6.91 ± 3.3 per 1000 inhabitants-year, respectively), the mean age of patients with stroke was significantly higher in women than in men (76.5 years ±12.3 vs 69.1 years ±13.2; *p* < 0.001).

**Fig. 2:**
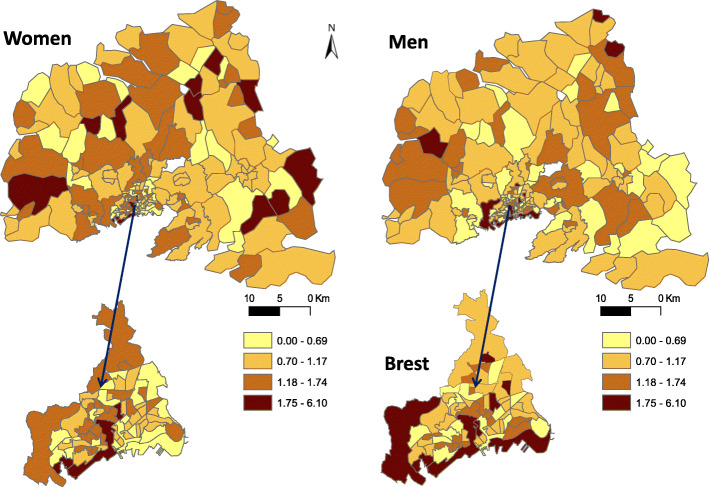
Age-standardized stroke incidence risk per 1000 inhabitants-year, in men and women living in the different census blocks of the Pays de Brest administrative region, France. Maps were created by the authors using ArcGIS software

**Fig. 3:**
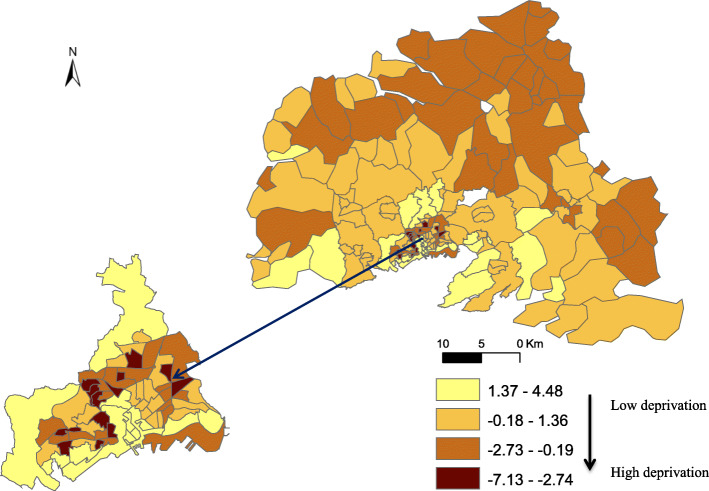
Spatial distribution of the deprivation level (FDep index) in the different census blocks within the Pays de Brest administrative region, France. The map was created by the authors using ArcGIS software

Stroke incidence in men seems higher mostly in census blocks located in the city of Brest and a few census blocks located far away from the city, in the northern part. Conversely, stroke incidence in women seems: showed no geographical pattern (Fig. 2). The FDep index indicated a south-north deprivation gradient, with lower deprived census blocks close to the littoral and the city of Brest, and more deprived blocks in rural and isolated areas (Fig. 3).

### Socioeconomic deprivation as a determinant of stroke incidence in women

First, the age-standardized stroke incidence risk was significantly higher for women living in high deprived and rural census blocks (Class 2) (1.27 [95%CI 1.09–1.43]) and in high deprived and urban census blocks (Class 4) (1.24, [95%CI 1.04–1.46]) than for those living in low deprived and urban census blocks (Class 3) (Table 1). Our study revealed no difference in terms of presence of comorbid cardiovascular disease(s) in women according to the level of deprivation of their census block (Table in Data Supplement). Moreover, stroke type (around 87% of ischemic strokes in women) and stroke severity were comparable in women whatever the deprivation level of their residential census blocks (around 17 and 12% of women had moderate/severe stroke, respectively) (Table in Data Supplement). The Koenker (BP) statistics showed a stationary relationship between stroke incidence risk and explanatory variables throughout the study area. For women, the negative binomial model with the deprivation & urban-rural index was the best model, showing the smallest AICc.

**Table 1:** Modeling the association between deprivation, urban-rural gradient, and stroke incidence

	Women Negative Binomial models		
	Coefficients	***p*** value	Goodness of fit
**FDep index**			
Intercept	0.002		
FDep index	−0.025	0.11	
AICc ^a^			883.1
Pseudo R-square value ^b^			1.5%
**Deprivation & Urban-rural index**			
Intercept	−0.08		
**Class 1:** Low deprived in suburban or isolated towns	0.016	0.82	
**Class 2:** High deprived in rural areas	0.237	**< 0.001**	
**Class 3:** Low deprived in urban areas	REF		
**Class 4:** High deprived in urban areas	0.212	**0.01**	
AICc ^a^			**872.3**
Pseudo R-square value ^b^			**7.9%**

^a^ Corrected Akaike Information Criterion often used for small number of observational data

^b^ R square value = percentage of deviance explained by the model

### Clusters of high stroke incidence risk in men

In contrast with women, there was in men evidence of spatial variations of the association between deprivation level and stroke risk. The Koenker (BP) statistics after the Poisson model indicated a statistically significant lack of stationarity. Therefore, the Poisson model was not appropriate, and GWR models were used. This was confirmed by the higher pseudo R^2^ value for the GWR models than for the Poisson models (R^2^ = 12.6% vs R^2^ = 0.5%) and the lower AICc for small samples (AICc = 593.8 vs AICc = 808.7, respectively).

**Fig. 4:**
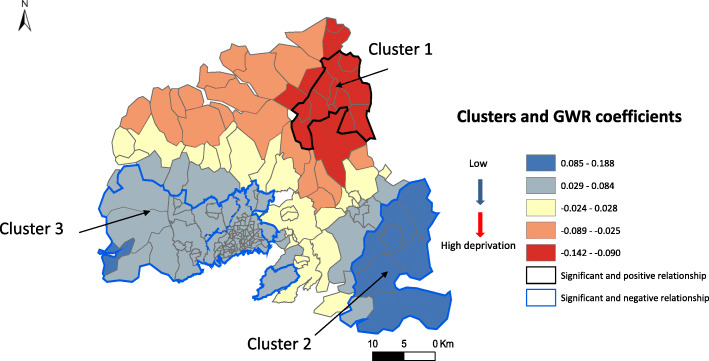
Clusters of higher risk of stroke incidence and deprivation map using GWR models. Negative deprivation scores indicate progressively higher deprivation levels, thus stroke incidence rate is negatively associated with high deprivation levels (negative scores) in Cluster 1 and positively associated with low deprivation levels (positive scores) in Clusters 2 and 3. The map was created by the authors using ArcGIS software

Three census block clusters with higher risk of stroke incidence were identified (Fig. 4). Cluster 1, in the northern and rural part of the Pays de Brest region (mainly class 2 census blocks), was associated with a 9–14% increase in the risk of stroke. In Cluster 2, located in the south-eastern part and composed of suburban, isolated and rural census blocks (classes 1 and 2), low deprivation level was associated with higher risk of stroke incidence (between 8.5 and 19%). In Cluster 3, located in the south-western part (Pays de Brest and Pays d’Iroise) and composed of urban and suburban census blocks, low deprivation was associated with higher risk of stroke incidence (between 3 and 8%).

### Comparison of male patient characteristics in the different clusters

The frequency of cardiovascular risk factors (hypertension, cardiac arrhythmia, diabetes, and dyslipidemia) was comparable among the three clusters and was similar to that of the rest of the study area (Table 2). No difference was observed concerning stroke type (> 88% of ischemic stroke cases in the three clusters and outside) and also stroke severity (29.9, 22.6 and 23.5% of patients with moderate/severe stroke in the three higher risk clusters; 26.5% in the rest of the study area; *p* = 0.49).

**Table 2:** Male patient characteristics in the three stroke clusters and in the rest of the study area

	Cluster 1	Cluster 2	Cluster 3	Rest of the study area
Number of patients within	***N*** = 57	***N*** = 31	***N*** = 644	***N*** = 588
**Sociodemographic features**				
Age (mean ± SD)	77.5 ± 8.1	74.4 ± 8.2	76.3 ± 8.6	76.3 ± 8.6
**Cardiovascular risk factors (%)**^b^				
Dyslipidemia	22.8	29.1	38.7	39.3
Diabetes	8.8	0	16.9	14.5
Cardiac arrythmia	28.1	12.9	23.5	25.9
High blood pressure	63.2	58.1	58.9	60.2
**Stroke type (%)**				
Ischemic	89.5	93.5	87.9	89.9
Hemorrhagic	10.5	6.5	11.7	10.1
**Stroke severity, NIHSS (%)**^a,b^				
Minor (0 to 5)	70.2	77.4	76.6	73.5
Moderate (6–13)	24.6	12.9	15.4	16.8
Severe (≥14)	5.3	9.7	8.1	9.7

^a^National Institute of Health Stroke Score (NIHSS)

^b^Missing values: 11% for severity and less than 4% for all patients regarding risk factors.

## Discussion

First, we have demonstrated some gender differences and specificities in stroke incidence. For women, the stroke incidence risk was homogeneous in the entire study area, whatever the degree of urbanization, but seemed to be influenced by the deprivation level. For men, three census block clusters with higher risk of stroke incidence were identified. The degree of urbanization and the deprivation level of the census blocks where men lived influenced the unequal distribution of stroke incidence. The incidence risk differences in men were not explained by the patients’ characteristics. Our study confirm that stroke affect men and women differently. A review from Wilson et al., examine the potential mechanisms underlying these differences and determine that there is a complex interaction between hormonal, genetic, and unknown factors [31].

For women, our study confirmed that the census block socioeconomic level explains a large portion of stroke incidence risk in women. Conversely, the stroke incidence risk was the same for women living in rural and urban areas. Census blocks with low socioeconomic level are characterized by lower education level and higher proportion of workers/farmers and of unemployment. Previous Swedish studies reported an increased risk of stroke in women with low annual income, but not in men [32], and a strong gradient in the stroke incidence risk in function of the years of education for women [33]. We could hypothesize that effect of local deprivation on stroke incidence risk in women is partly mediated by the higher prevalence of health-damaging behaviors, particularly cigarette smoking, poor dietary habits, sedentary lifestyle, obesity and alcohol [8], and reduced use of healthcare services in deprived neighborhoods [34]. Although it is known that medically oriented diet changes [35] and the consumption of healthy food are beneficial, awareness/implementation of such changes may be more challenging in deprived neighborhoods. Whereas the stroke incidence risk seemed to be influenced by the deprivation level of their census block, the presence of comorbid cardiovascular disease(s), stroke type and stroke severity, were similar according to the level of deprivation (Table in Data Supplement). Moreover, previous studies have investigated and demonstrated the possible strategy for stroke prevention of postmenopausal hormone replacement therapy (HRT) [36]. After the menopause, the incidence of these entities increases and this is related, among other causes, to the loss of the protective effect exerted by estrogens. Women with higher socioeconomic status are more likely to use hormone therapy with healthier lifestyles [37].

In our study, location of residence was a strong determinant of stroke incidence risk for men, and three clusters with high stroke incidence were detected. Stroke incidence was higher for men located in a rural cluster composed mostly of deprived census blocks (Cluster 1). Previous studies demonstrated that rural and lower socioeconomic populations face barriers, such as shortages of healthcare professionals, lack of health insurance, transportation difficulties, later presentation, poor uptake of preventive or screening procedures, disparities in knowledge of risk reduction practices and geographic distance [38]. Dobson et al. concluded that people in rural areas may be exposed to a double disadvantage: poor health services and higher exposure to health hazards [38]. Previous studies have shown that social or geographic ‘barriers’ are likely to contribute towards access to a specialist care unit [39].

The risk of stroke incidence was higher also for men living in suburban and urban census blocks in the southern part of Pays de Brest (clusters 2 and 3). The environmental profiles of these census blocks are quite heterogeneous. Cluster 3 is composed by some urban census blocks close to the specialist care unit in the city of Brest but these are mostly deprived with higher proportions of workers, higher unemployment rates, and lower education levels. Cluster 2 was composed mostly of rural and census blocks located in pays de Landerneau. We suppose that location of residence in rural areas is a strong determinant of easy access to healthcare and use of healthcare services.

Finally, our study did not find any difference in the presence of comorbid cardiovascular disease and in stroke severity in men within the three clusters. A recent study in Pays de Brest reported similar results: risk factors and stroke type and severity were comparable among urban categories in both sexes [40]. An interpretation of these findings is that a similar combination of risk factors led to stroke occurrence within and outside clusters. Whether risk factors prevalence varies in the underlying populations, can only be explored on representative sample of the entire (affected and non-affected) population.

Local Poisson GWR models can be a valuable tool to explore the complex relationships between stroke (or other chronic diseases) and risk factors, when previous studies demonstrated spatial variability [20]. Using ordinary models for women, we found that the stroke incidence was equally distributed in the study area, whatever the degree of urbanization or the level of deprivation of the census blocks. For men, the stroke incidence was not equally distributed, suggesting that ordinary Poisson models are less accurate and reliable and would provide bias results. GWR models bring significant improvement taking into account the presence of non-stationarity [20].

The main strength of our study was the use of the Brest Stroke Registry to identify all case of stroke in the Pays de Brest administrative region. This registry was validated by source analysis and the capture-recapture method, and is the most relevant tool to study the epidemiology of stroke because it ensures the data quality and exhaustiveness [26]. Moreover, small area analysis (census block) allows a deeper understanding of the geographic patterns of health inequalities, and is essential for revealing local-level inequalities that are often masked when health estimates are produced at larger area scales (cities, countries, and states). Analysis at the census block level allowed a better definition of the spatial patterns of stroke incidence risk. Another strength of our approach was the production of a map with areas of significantly higher risk of stroke incidence that can be used for future interventions and surveillance.

Our study presents also some weaknesses. High stroke risk has been attributed to variations in the distribution of cardiovascular risk factors, as race, socioeconomic status, geography (urban and rural). Unfortunately, our study was not adjusted for variations in other chronic diseases and race. As some previous studies suggested the role of nutraceuticals in cardiovascular risk protection, information on the diet habits could have been used to investigate this relation. Moreover, the lower socioeconomic status of rural populations may influence their access to quality healthcare, and we did not have any data on this point. Similarly, we did not have any individual data on traditional risk factors, such as physical inactivity, cholesterol level and pharmacological treatments, or on the genetic risk of cardiovascular disease.

Future studies could focus on all dimensions of healthcare access, such as distance from the hospital, accessibility of stroke units, regular and easy access to primary and specific healthcare. As controlling the lipid levels can reduces cardiovascular risk in individuals with dyslipidemia **[35],** the intake of pharmacological treatments and nutraceuticals should be evaluated. For women, further studies need to analyse the patterns of use of hormone replacement therapy. Moreover, more research should be done on modifiable factors related to beliefs, diets and habits to improve stroke prevention in Pays de Brest.

## Conclusions

In conclusion, we found geographic variations in stroke incidence risk in the Pays de Brest administrative region, France. Our study confirms that for women, the highest rates were in the most deprived census blocks, independently of their degree of urbanization. For men, we identified three clusters of higher risk of stroke incidence: one cluster in the north (census blocks that combine rurality and socioeconomic deprivation), and two clusters in the south with heterogeneous environmental (urbanization and socioeconomic) patterns, but lower distance and higher accessibility to stroke units. These geographic differences are not explained by differences in the patients’ characteristics. This finding suggests that other factors. The implication of this study would be to improve the recommendation in prevention regarding the easily modifiable factors that could decrease the incidence of major chronic disease, as well as access to care, use of treatments, hormone replacement therapy. Understanding whether and how neighborhood and patient’s characteristics influence stroke risk may be useful for both epidemiological research and healthcare service planning.

## Supplementary Information

**Additional file 1 Table.** Women patients stroke risk factors, stroke type and severity according to the level of deprivation of their residential census blocks

## Data Availability

The access to Brest stroke registry data is regulated by a scientific committee, which analyzes each request. In this context, data available upon request. If readers need information about the data, they can contact Pr. Serge Timsit (who coordinates the Stroke registry).

## Abbreviations

AICc: Corrected Akaike Information Criterion
FDep: French deprivation
GWR: Geographically weighted regression
INSEE: National Institute of Statistics and Economic Studies
NIHSS: National Institute of Health Stroke Score
PCA: Principal component analysis
SES: Socioeconomic status
