# Supplementary material for: Gender difference of geographic distribution of the stroke incidence affected by socioeconomic, clinical and urban-rural factors: an ecological study based on data from the Brest stroke registry in France
Source: BMC Public Health. 2021 Jan 6;21:39. doi: 10.1186/s12889-020-10026-7 (PMC7788878; doi:10.1186/s12889-020-10026-7)
Supplement: Supplementary file 1 — Additional file 1 Table. Women patients stroke risk factors, stroke type and severity according to the level of deprivation of their residential census blocks [file 12889_2020_10026_MOESM1_ESM.docx]

**Table:** Women patients stroke risk factors, stroke type and severity according to the level of deprivation of their residential census blocks

|  |  | High deprived Census Blocks | Middle deprived Census blocks | Low deprived Census blocks | p values * |
| --- | --- | --- | --- | --- | --- |
| Number of patients within | | N=531 | N=577 | N=648 |  |
| **Risk factors (%)** | |  |  |  |  |
| Dyslipidemia | | 32.2 ± 4.6 | 33.8 ± 4.7 | 30.7 ± 4.6 | 0.65 |
| Diabetes | | 13.5 ± 3.4 | 11.4 ± 3.2 | 12.8 ± 3.3 | 0.55 |
| Cardiac arythmia | | 25.6 ± 4.4 | 24.1 ± 4.3 | 26.8 ± 4.4 | 0.71 |
| Hypertension | | 68.5 ± 4.6 | 71.4± 4.5 | 71.7± 4.5 | 0.43 |
|  |  |  |  |  |  |
| **Stroke type (%)** | |  |  |  |  |
| Ischemic | | 87.2 | 85.6 | 87.2 | 0.66 |
| Hemorrhagic | | 12.8 | 14.4 | 12.8 |  |
|  |  |  |  |  |  |
| **Stroke severity NIHSS (%)** | |  |  |  |  |
| Low (0 to 5) | | 67.4 | 67.4 | 70.2 | 0.45 |
| Medium (6 to 13) | | 19.8 | 17.3 | 17.1 |  |
| High (14 or more) | | 12.8 | 15.2 | 12.6 |  |

*p values according to Kruskall Wallis tests
